# Increased sputum peripheral helper T cells are associated with the severity of rheumatoid arthritis but not with the severity of airway disease

**DOI:** 10.3389/fimmu.2025.1526881

**Published:** 2025-02-27

**Authors:** Eito Yokoi, Keiko Wakahara, Saya Nakamura, Eriko Fukutani, Shuji Asai, Nobunori Takahashi, Toshihisa Kojima, Shingo Iwano, Satoko Shimada, Toyofumi Fengshi Chen-Yoshikawa, Naozumi Hashimoto, Makoto Ishii

**Affiliations:** ^1^ Department of Respiratory Medicine, Nagoya University Graduate School of Medicine, Nagoya, Japan; ^2^ Department of Orthopedic Surgery and Rheumatology, Nagoya University Graduate School of Medicine, Nagoya, Japan; ^3^ Department of Orthopedic Surgery, Aichi Medical University, Nagakute, Japan; ^4^ Department of Orthopedic Surgery, National Hospital Organization Nagoya Medical Center, Nagoya, Japan; ^5^ Department of Radiology, Nagoya University Graduate School of Medicine, Nagoya, Japan; ^6^ Department of Pathology, Nagoya University Hospital, Nagoya, Japan; ^7^ Department of Thoracic Surgery, Nagoya University Graduate School of Medicine, Nagoya, Japan; ^8^ Department of Respiratory Medicine, Fujita Health University, Toyoake, Japan

**Keywords:** rheumatoid arthritis, peripheral helper T cells, sputum, lung tissue, airway disease

## Abstract

**Objective:**

Peripheral helper T (Tph) cells, together with plasma cells, are the major pathogenic lymphocytes in the synovium in rheumatoid arthritis (RA). However, whether these cells are involved in RA-associated lung and/or airway disease is unknown.

**Methods:**

Tph cells in sputum were analyzed by flow cytometry and compared with those in synovial fluid and synovial tissue. Forty RA subjects for whom induced sputum could be collected were analyzed along with sputum Tph cells and several clinical parameters; RA severity was assessed using the Disease Activity Score for 28 joints (DAS28). Lung and airway disease was assessed by chest computed tomography (CT), pulmonary function test, the chronic obstructive pulmonary disease (COPD) Assessment Test (CAT), and sputum culture. Tph cells in the lung of RA subjects were analyzed using lung resection samples in a separate cohort.

**Results:**

Tph cells were observed in the sputum, as well as the lung, synovial fluid, and synovial tissue of RA patients. Sputum Tph cells were increased in patients with airway disease. Among these patients, Tph cells were more frequent in those with high DAS28, high serum immunoglobulin G (IgG), and high sputum IgG. However, there was no association between Tph cells and the severity of airway disease as assessed by chest CT findings, lung function, CAT, and sputum culture.

**Conclusions:**

Tph cells were increased in the airways as well as in the synovium in patients with RA. Airway Tph cells were associated with severity of RA but not with the severity of airway disease. Airway Tph cells may represent a novel target for disease management and treatment.

## 1 Introduction

PD-1^hi^CXCR5^-^CD4^+^ peripheral helper T (Tph) cells have attracted attention as pathological T cells in rheumatoid arthritis (RA) patients. Originally, these cells were found in the joint tissues of RA patients and were found to have B helper-associated functions such as plasma cell differentiation (1). Tph cells are increased in the blood of seropositive RA patients, and their numbers reflect disease severity and response to treatment (1–3). The involvement of Tph cells in other autoimmune diseases has been demonstrated by several groups (4, 5). Circulating Tph cells have been observed in systemic lupus erythematosus (SLE) (6, 7), Sjogren’s syndrome (8–10), IgG4-related disease (8), type 1 diabetes (11), primary biliary cirrhosis (12), immunoglobulin A nephropathy (13), juvenile idiopathic arthritis (14), autoimmune hepatitis (15), autoimmune bowel disease (16), and psoriasis vulgaris (17). They have also been shown to be associated with disease activity and are a potential clinical biomarker and therapeutic target. However, analyses of Tph cells in inflamed tissues are scarce.

Although RA is a chronic, systemic autoimmune inflammatory disorder that mainly affects the joints and periarticular soft tissues, lung and airway diseases (AD) are known to be major extra-articular complications (18). Symptomatic interstitial lung disease (ILD) and AD occur in 5–17% and up to 30% of RA patients, respectively. However, when radiographic abnormalities are included, their frequency reaches 30% and 60%. Sometimes lung disease appears to precede the onset of joint symptoms (19). In addition, the fact that anti-citrullinated protein antibodies (ACPA) are produced in the lungs years before the onset of articular symptoms (20) suggests the involvement of lung/airway mucosal sites in disease modification.

Here, we hypothesized that the topical Tph cells are also present in the lungs/airways of RA patients and assessed them in sputum and lung specimens from RA patients. We then analyzed associations with other immune cells, laboratory data, RA severity, and airway disease severity to determine the clinical impact of these cells.

## 2 Materials and methods

### 2.1 Subjects

Fifty-one adult patients with RA were recruited from the outpatient clinic of Nagoya University Hospital from April 2019 to March 2021. All patients had been diagnosed with RA by rheumatologists, and met the 1987 American College of Rheumatology (ACR) classification criteria (21) or the new ACR/European League Against Rheumatism (EULAR) diagnostic criteria (22). Of the 51 patients, the sputum of 40 patients who were able to provide it was analyzed (**Supplementary Figure S1**). Controls were healthy subjects with no history of smoking (n=6). Patients with active infection requiring additional treatment, respiratory failure, or malignant disease were excluded. For the analysis of synovial fluid, synovial tissues, and lung specimens, RA patients who underwent surgery at our hospital were recruited as separate cohorts from the main cohort. All subjects signed informed consent forms approved by the Ethics Committee of Nagoya University (approval numbers 2012-0078 for sputum and lung analysis and 2020-0454 for synovial fluid and synovial tissue analysis).

### 2.2 Data collection and measurement of disease activity

Patient clinical information was obtained from the medical record and included tender joint count, swollen joint count, and patient global assessment on the day of sample collection. The standard blood test was carried out in the laboratory of our hospital. Disease activity was evaluated using the 28-joint disease activity score (DAS28) with erythrocyte sedimentation rate (ESR) and DAS28 with C-reactive protein (CRP).

### 2.3 Assessment of lung and airway diseases

For the ILD and AD assessment, spirometry analysis, modified medical research council dyspnea scale (mMRC), chronic obstructive pulmonary disease (COPD) assessment test (CAT), measurement of exhaled nitric oxide fraction (FeNO; NiOX Vero Aerocrine, Solna, Sweden), standard sputum culture, sputum acid-fast bacilli culture, and chest high-resolution computed tomography (HRCT) were performed. Interstitial pneumonia, granular shadow, bronchiectasis, and mucus plugs were evaluated and scored by a pulmonologist and radiologists as indicated in **Supplementary Table S1** and **Supplementary Figure S2**.

AD patients were defined as patients who have shown granular shadow(s) and/or bronchiectasis on chest HRCT, asthma, or COPD. ILD patients were defined as patients showing interstitial pneumonia on chest HRCT.

### 2.4 Blood and sputum collection and processing

Peripheral blood mononuclear cells (PBMCs) were prepared using BD Vacutainer CPT Tubes (Becton Dickinson and Co., Franklin Lakes, NJ, USA). Sputum samples were obtained using an established protocol as previously reported (23). Briefly, the inhalation of nebulized 5% saline for 5 min was conducted up to five times. The samples were weighed, treated with two volumes of 0.05% dithiothreitol (DTT; Wako Pure Chemical Industries, Ltd., Osaka, Japan), and incubated for 30 min at 37°C. The suspension was then filtered through a 70-μm nylon cell strainer to remove cell debris and mucus. Sputum inflammatory cells were evaluated by microscopic examinations after Diff Quick staining (Sysmex, Kobe, Japan).

### 2.5 Tissue sample collection and processing

Synovial samples (fluid and tissue) were obtained as excess material from a separate cohort of patients undergoing arthroplasty (**Supplementary Table S2**). Lung samples were obtained from areas of resected specimens from lung cancer patients sufficiently distant from the cancer site (**Supplementary Table S3**). Synovial and lung tissues were cut into small pieces with scissors, digested with collagenase D and DNase I(Roche Applied Science, Penzberg, Germany), and dissociated by a gentle MACS dissociator (Miltenyi Biotec, North Rhine-Westphalia, Germany) (24). After filtration through a 70-μm nylon cell strainer, the remaining red blood cells were lysed with ACK lysing buffer. Histopathology of the lung tissue was evaluated by hematoxylin and eosin (H&E) staining and immunohistochemistry (IHC). IHC staining was performed using BOND-III (Leica Biosystems, Nussloch, Germany) with anti-CD3 (clone LN10, Leica Biosystems, dilution 1:400), anti-CD20 (clone L26, Leica Biosystems, dilution 1:400), or anti-CD138 (clone MI15, DAKO, Glostrup, Denmark, dilution 1:800) in the hospital laboratory.

### 2.6 Flow cytometry analysis

After blocking with Human TruStain FcXTM (BioLegend, San Diego, CA, USA), cells were stained with anti-CD3-FITC, anti-CD4-PE/Cy7, anti-CD14-BV510, anti-CD19-PerCP, anti-CD38-PE, anti-CD45RA-PerCP, anti-CD138-APC, anti-CD185 (CXCR5)-APC, anti-CD192 (CCR2)-BV421, anti-CD279 (PD-1)-PE (BioLegend), and anti-CD45-APC-H7 (BD Bioscience, San Jose, CA, USA) and fixed. Cells were analyzed using FACS Canto (Becton, Dickinson and Co., Franklin Lakes, NJ, USA), and data were analyzed using FlowJo software (FlowJo LLC, Ashland, OR, USA). Tph cells were defined as CD45^+^CD3^+^CD4^+^CD14^-^CD45RA^-^PD-1^hi^CXCR5^-^cells, B cells were defined as CD45^+^CD3^-^CD4^-^CD14^-^CD19^+^ cells, and plasma cells were defined as CD45^+^CD3^-^CD4^-^CD14^-^CD19^+^CD38^+^CD138^+^cells. Fluorescence spillover into the PE channel was assessed by fluorescence minus one (FMO) control (**Supplementary Figure S3**).

### 2.7 Statistical analysis

Data were analyzed by SPSS version 28 (SPSS, Inc., Chicago, IL, USA). Results are expressed as mean values ± SEM. Comparisons between the two groups were performed using the non-parametric Mann-Whitney test, Pearson’s chi-square test, or Fisher’s exact test. Correlation was evaluated by the Spearman rank test. P-values <0.05 were considered statistically significant.

## 3 Results

### 3.1 Tph cells were detectable in sputum of RA patients

**Figure 1:**
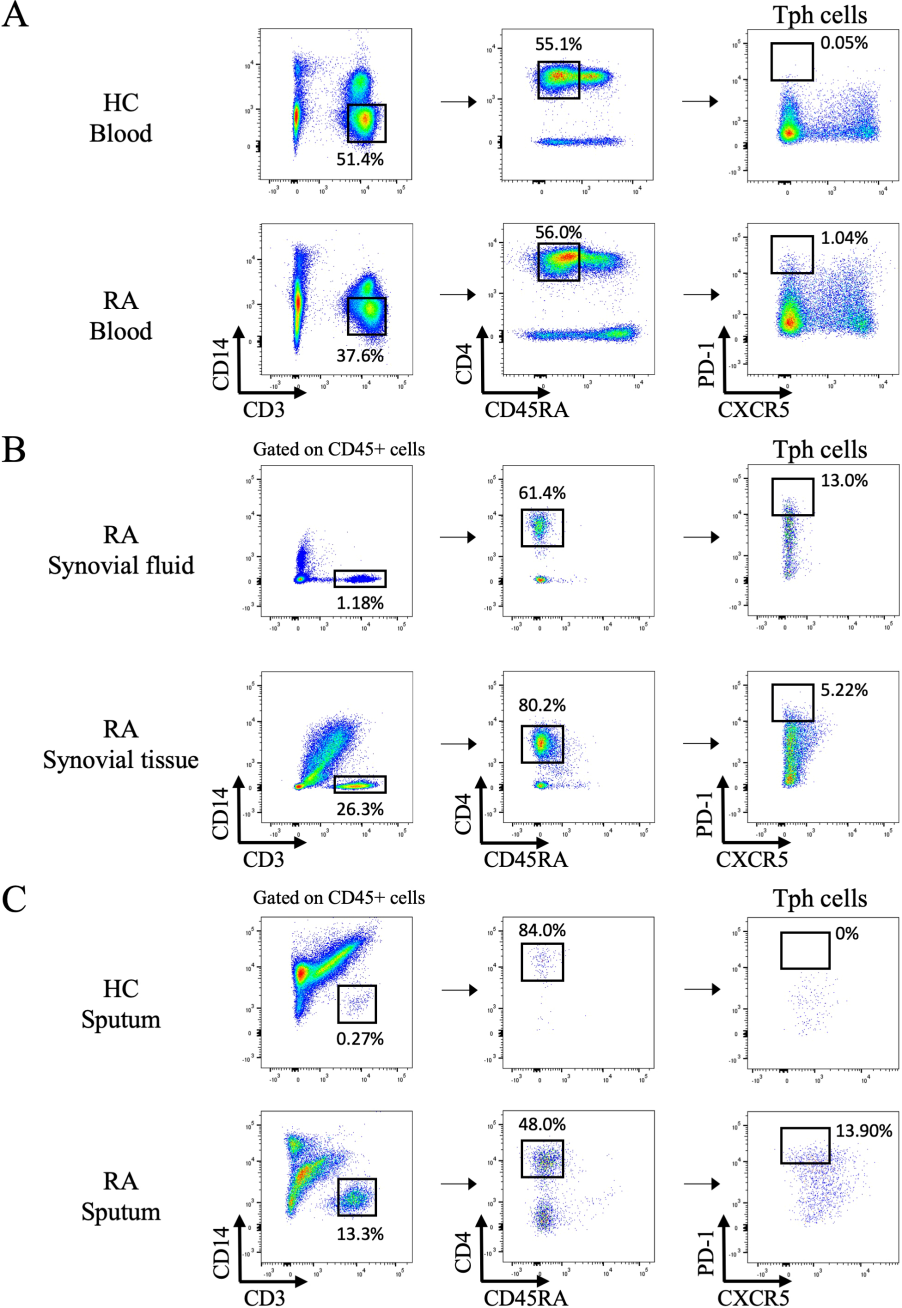
Flow cytometry analysis of peripheral helper T cells in the blood, synovial fluid, synovial tissue, and sputum. **(A)** Gating strategy to identify peripheral helper T (Tph) cells among peripheral blood mononuclear cells isolated from healthy controls **(HC)** and rheumatoid arthritis (RA) patients by flow cytometry. Representative data of 6 HC and 38 RA patients. **(B)** Tph cells in synovial fluid and synovial tissue from RA patients. Representative data of 5 samples each. **(C)** Tph cells in sputum from HC and RA patients. Representative data from 6 HC and 40 RA samples.

We first confirmed the frequency of Tph cells in the peripheral blood, synovial fluid, and synovial tissue using flow cytometry. As previously reported, PD-1^hi^ CXCR5^-^ CD4^+^ cells were observed in RA patients (**Figures 1A, B**). In addition, Tph cells were detectable in sputum of RA patients using the same gating strategy as for synovial fluid and synovial tissue (**Figure 1C**, **Supplementary Figure S4**).

### 3.2 Sputum Tph cells were increased in RA patients with airway disease

**Figure 2:**
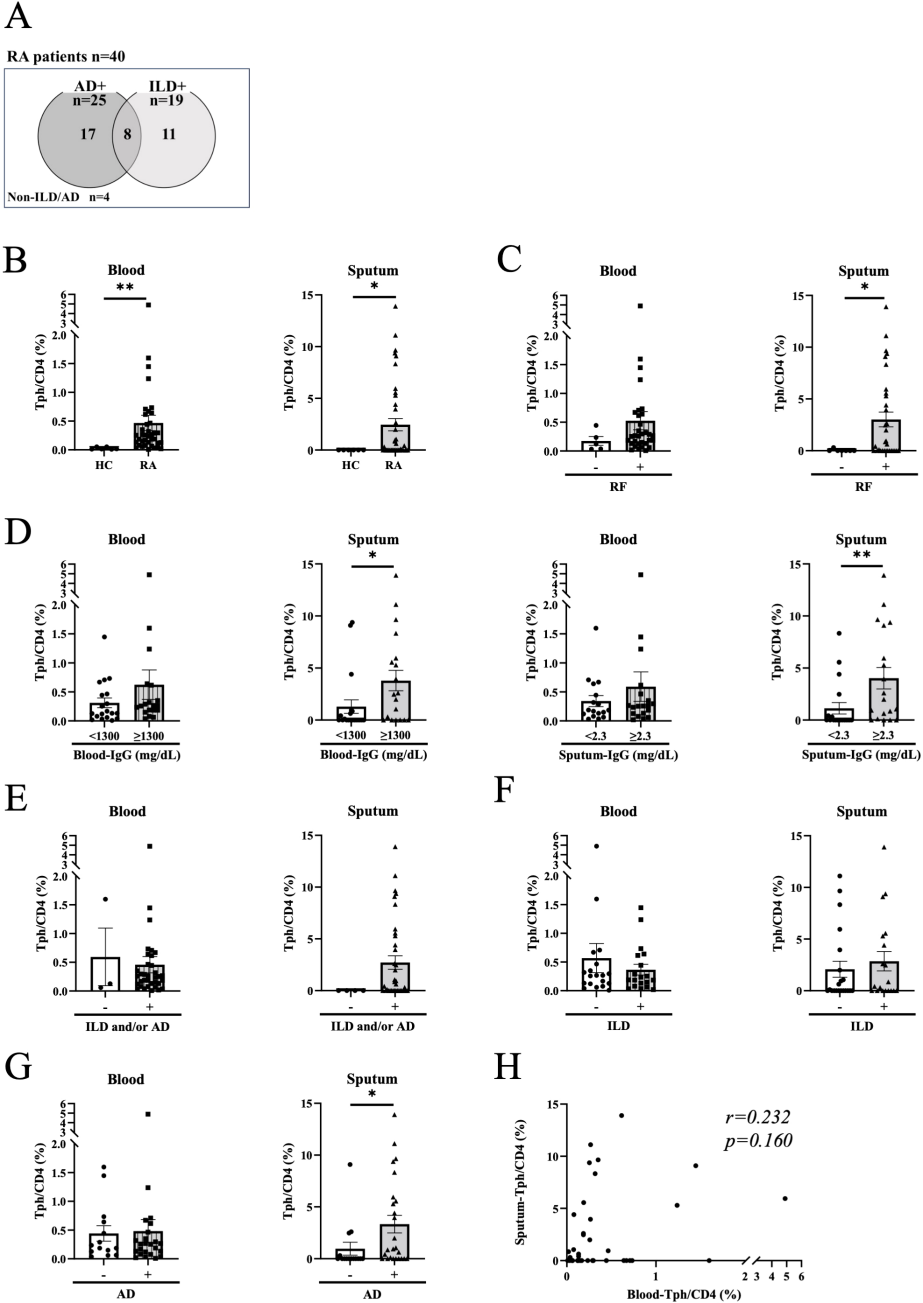
Blood and sputum peripheral helper T cells in rheumatoid arthritis patients. **(A)** Rheumatoid arthritis (RA) patients included in the analysis. Venn diagram showing the overlap of airway disease (AD) and interstitial lung disease (ILD) in RA patients. Non-ILD/AD indicates RA without apparent lung/airway disease. **(B)** Peripheral helper T (Tph) cells in blood and sputum from healthy controls (HC) (n=6) and RA patients (n=38-40). **(C, D)** Tph cells from RA patients divided by the positive (n=32) or negative (n=5-7) for rheumatoid factor (RF) **(C)**, high or low for blood IgG levels (high n=19-20, low n=19) and sputum IgG levels (high n=17-19, low n=19) **(D)**. Blood and sputum IgG cutoff values were determined by the median of all specimens. **(E–G)** Tph cells from RA patients divided by the presence or absence of lung disease (ILD and/or AD) (presence n=35-36, absence n=3-4) **(E)**, ILD (presence n=19, absence n=19-21) **(F)**, or AD (presence n=24-25, absence n=14-15) **(G)**. **(H)** Correlation between blood Tph cells and sputum Tph cells in RA patients (n=38) evaluated by Spearman’s correlation coefficient. Data are expressed as mean ± SEM. **(B–G)** *P<0.05, **P<0.01 determined by Mann-Whitney test.

To address the clinical relevance of sputum Tph cells, we next examined the relationship between blood and sputum Tph cells, and clinical parameters of RA patients. Characteristics of the patients are shown in **Table 1**. Among 40 patients of the analysis target, 25 had AD and 19 had ILD, with some overlap (**Table 1**, **Figure 2A**). The mean disease duration was 12.74 years, and 95% of patients had been treated with disease-modifying anti-rheumatic drugs (DMARDs) and/or glucocorticoids. Disease activity as assessed by the DAS 28 ESR was as follows: remission, low, moderate, and high were 8, 8, 15, and 2 patients, respectively. **Figure 2** shows the frequencies of Tph cells in the blood and sputum of all RA patients analyzed (n=40). Tph cells in blood and sputum were significantly higher in RA patients than in healthy controls (**Figure 2B**). Sputum Tph cells were significantly more numerous in patients with positive for rheumatoid factor (RF), high blood IgG levels, and high sputum IgG levels, but no such difference was observed for blood Tph cells (**Figures 2C, D**). There was no difference in the frequency of Tph cells in the blood and sputum between patients with positive and negative anti-CCP2 antibodies (**Supplementary Figure S5**). Sputum Tph cells tended to increase in RA patients with ILD and/or AD and were significantly increased in patients with AD but not in patients with ILD (**Figures 2E–G**). However, this was not the case in blood, and there was no correlation between sputum Tph cells and blood Tph cells in RA patients (**Figure 2H**).

**Table 1:** Study population.

	All	AD (-)	AD (+)	ILD (-)	ILD (+)
Patients, n	40	15	25	21	19
Age, median (range) years	71 (35-82)	70 (37-79)	71 (35-82)	69 (35-82)	72 (51-79)
Female, n (%)	27 (67.5)	9 (60.0)	18 (72.0)	19 (90.5)	8 (42.1) ^†^
Duration of RA (years)	12.74 ± 9.02	12.80 ± 8.64	12.71 ± 9.44	14.05 ± 9.89	11.22 ± 7.90
Smoking history (never/ex/current), n	22/15/3	2007/7/1	2015/8/2	2015/5/1	2007/10/2
Pack-years	21.3 ± 35.37	22.12 ± 32.92	20.83 ± 37.41	5.69 ± 15.13	38.58 ± 43.09^††^
CAT score, median (range)	10 (2-27)	8 (2-20)	10 (2-27)	9 (2-27)	10 (2-18)
mMRC score (0/1/2/3/4), n	9/20/9/1/1	2/9/4/0/0	7/15/5/1/1	6/12/1/1/1	3/8/8/0/0
DAS28-CRP	2.57 ± 1.04	2.54 ± 0.82	2.60 ± 1.17	2.58 ± 0.77	2.57 ± 1.31
DAS28-ESR	3.32 ± 1.11	3.30 ± 0.86	3.34 ± 1.27	3.26 ± 0.76	3.38 ± 1.42
Disease activity (remission/low/moderate/high), n	8/8/15/2	3/3/6/1	5/5/9/1	4/4/9/0	4/4/6/2
RF (positive/negative), n	32/7	11/4	21/3	17/3	15/4
ACPA (positive/negative), n	35/3	14/0	21/3	16/3	19/0
Treatment characteristics					
Use of oral glucocorticoids, n (%)	17 (42.5)	6 (40)	11 (44.0)	5 (23.8)	12 (63.2) ^†^
Use of methotorexate, n (%)	18 (45.0)	5 (33.3)	13 (52.0)	11 (52.4)	7 (36.8)
Use of bDMARDs, n (%)	13 (32.5)	7 (46.7)	6 (24.0)	6 (28.6)	7 (36.8)
Chest CT					
Pattern (normal/ILD/airway lesion/mixed)	5/16/16/3	4/11/0/0	1/5/16/3	5/0/16/0	0/16/0/3
ILD, n (%)	19 (47.5)	11 (73.3)	8 (32.0) ^*^	0	19 (100)
Bronchiectasis, n (%)	17 (42.5)	0	17 (68.0)	15 (71.4)	2 (10.5) ^†††^
Granular shadow, n (%)	16 (40)	0	16 (64.0)	14 (66.7)	2 (10.5) ^†††^
Lung function test					
VC % predicted (%)	96.79 ± 16.13	96.42 ± 13.38	97.03 ± 17.98	101.96 ± 17.24	91.61 ± 13.44^†^
FEV_1_% predicted (%)	95.19 ± 18.25	99.08 ± 13.58	92.66 ± 20.64	100.25 ± 20.78	90.13 ± 14.10^†^
FEV_1_/FVC % (%)	75.64 ± 8.42	79.89 ± 6.70	72.86 ± 8.39^*^	74.94 ± 10.56	76.33 ± 5.78
Complications					
Asthma, n (%)	5 (12.5)	0	5 (20)	3 (14.3)	2 (10.5)
COPD, n (%)	5 (12.5)	0	5 (20)	1 (4.8)	4 (21.1)
Bacteria (sputum culture), n (%)	15 (37.5)	3 (20)	12 (48.0)	11 (52.4)	4 (21.1) ^†^
NTM (sputum culture), n (%)	9 (22.5)	0	9 (36) ^**^	7 (33.3)	2 (10.5)

AD, Airway disease; ILD, Interstitial lung disease; mMRC, Modified Medical Research Council Dyspnea Scale; CAT score, COPD Assessment Test score; DAS28, 28-joint disease activity score; RF, rheumatoid factor; ACPA, anti-citrullinated protein antibodies; bDMARDs, biological disease-modifying antirheumatic drugs; VC, vital capacity; FEV_1_, forced expiratory volume in one second; COPD, chorionic obstructive pulmonary disease; NTM, nontuberculous mycobacteria.

Disease activity was classified based on DAS28-ESR (remission, <2.6; low disease activity, ≥2.6 and <3.2; moderate disease activity, ≥3.2 and ≤5.1; and high disease activity, >5.1).

AD (+) included 8 ILD-combined patients.

ILD (+) included 8 AD-combined patients.

Data presented as n (%) or mean ± SD unless otherwise noted.

^*^P<0.05, ^**^P<0.01 vs. the AD (-), determined by Mann-Whitney U test, Pearson’s chi-square test, or Fisher’s exact test

^†^P<0.05, ^††^P<0.01, ^†††^P<0.001, vs. the ILD (-), determined by Mann-Whitney U test, Pearson’s chi-square test, or Fisher’s exact test

### 3.3 Sputum Tph cells were increased in RA patients with high disease severity

**Figure 3:**
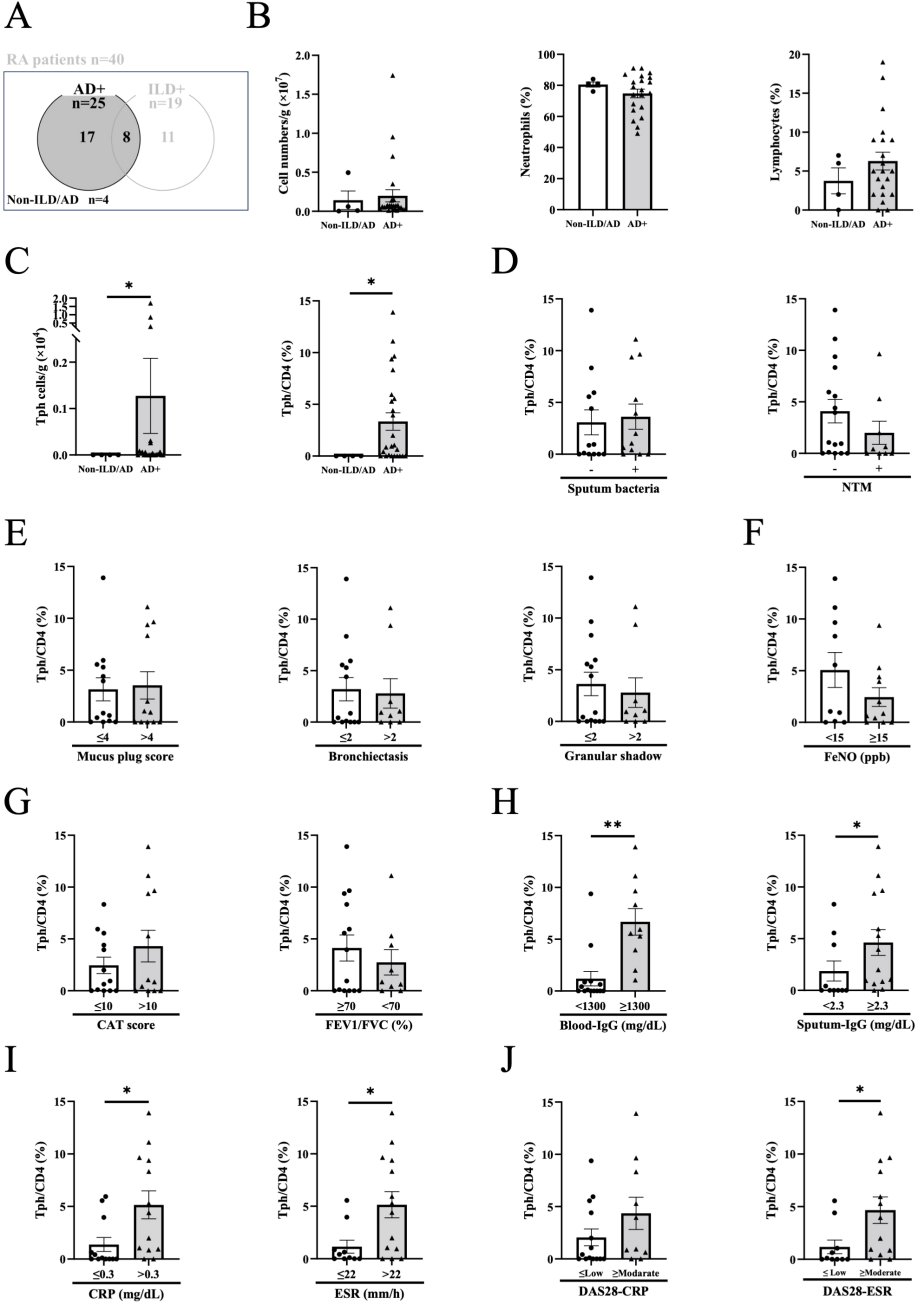
Peripheral helper T cells in airway disease. **(A)** Sputum from rheumatoid arthritis (RA) patients with airway disease (AD) in the presence or absence of interstitial lung disease (ILD) was evaluated. Non-ILD/AD indicates RA without apparent lung/airway disease. **(B)** Total cell counts, percentages of neutrophils and lymphocytes in the sputum of RA patients with AD (Non-ILD/AD n=4, AD n=21-25). **(C)** Total number and percentage of peripheral helper T (Tph) cells from the sputum of RA patients with AD (Non-ILD/AD n=4, AD n=23-25). **(D)** Sputum Tph cells based on the positive or negative general sputum culture (sputum bacteria) (positive n=12, negative n=13) and acid-fast bacilli culture (nontuberculous mycobacteria: NTM) (positive n=9, negative n=16). **(E–I)** Sputum Tph cells divided by chest computed tomography (CT) findings (plugging (≤4 n=13, >4 n=12), bronchiectasis (≤2 n=14, >2 n=9), and granular shadow (≤2 n=15, >2 n=9)) **(E)**, the value of exhaled nitric oxide fraction (FeNO) (<15 ppb n=10, ≥15ppb n=11) **(F)**, COPD assessment test (CAT) score (≤10 n=13, >10 n=12) and FEV_1_/FVC% (≥ 70% n=14, <70% n=9) **(G)**, blood IgG levels (<1300 mg/dL n=14, ≥ 1300 mg/dL n=10) and sputum IgG levels (<2.3 mg/dL n=10, ≥ 2.3 mg/dL n=14) **(H)**, CRP (≤0.3 mg/dL n=12, >0.3 mg/dL n=13) and ESR (≤22 mm/h n=10, >22 mm/h n=14) **(I)**, and DAS-CRP (≤Low n=14, ≥Moderate n=10) and DAS28-ESR (≤Low n=10, ≥Moderate n=13). Disease activity based on DAS28-CRP (remission, <2.3; low disease activity, ≥2.3 and <2.7; moderate disease activity, ≥2.7 and ≤4.1; and high disease activity, >4.1) and on DAS28-ESR (remission, <2.6; low disease activity, ≥2.6 and <3.2; moderate disease activity, ≥3.2 and ≤5.1; and high disease activity, >5.1) **(J)**. Blood and sputum IgG and FeNO cutoff values were determined by the median of all subjects. Data are shown as mean ± SEM. *P<0.05, **P<0.01 determined by Mann-Whitney test.

Because sputum Tph cells were found to be elevated in RA patients with AD, we focused on the impact of sputum Tph cells in AD (**Figure 3A**, n=25). Although the total number of cells, the percentages of neutrophils and lymphocytes in sputum cells with AD were not increased compared to those without ILD/AD, the number and percentage of Tph cells were significantly increased in AD (**Figures 3B, C**). Within AD, there were no differences in sputum Tph cells according to general sputum bacterial culture and acid-fast bacilli (for nontuberculous mycobacteria: NTM) culture results (**Figure 3D**), CT findings (**Figure 3E**), FeNO (**Figure 3F**), symptoms (CAT score), or lung function (**Figure 3G**). However, sputum Tph cells were increased in patients with high blood IgG, high sputum IgG, high CRP, and high ESR (**Figures 3H, I**). In addition, the frequency of sputum Tph cells was significantly correlated with blood IgG and sputum IgG levels (**Supplementary Figure S6**). Furthermore, sputum Tph cells tended to be increased in patients with high DAS 28-CRP and were increased in patients with high DAS 28-ESR (**Figure 3J**). These findings were also observed when excluding AD patients with ILD (n=17) (**Supplementary Figure S7**).

### 3.4 Frequency of Tph cells and B cells correlated in the lung tissue and sputum of RA patients

**Figure 4:**
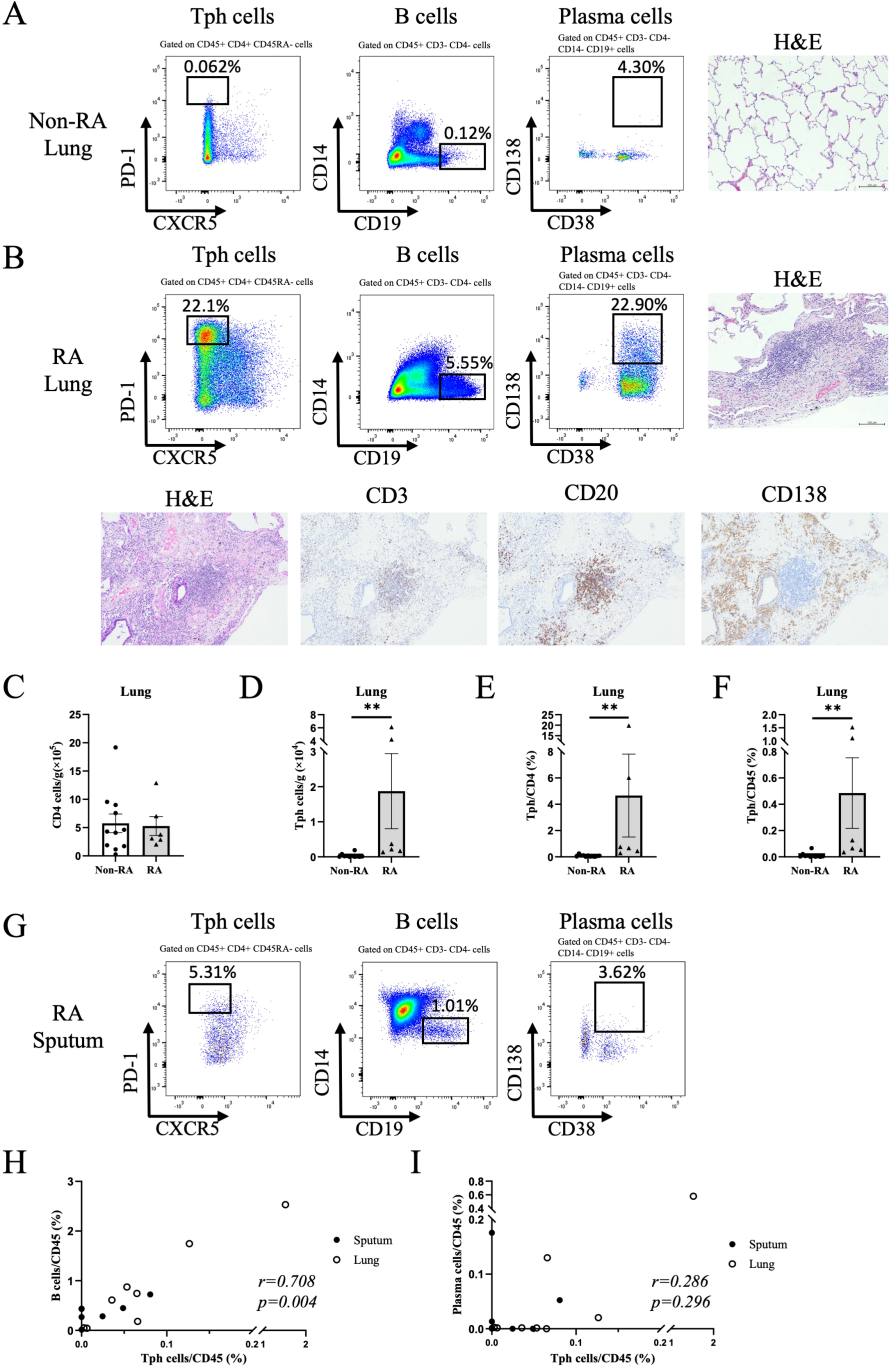
Flow cytometry analysis of peripheral helper T cells, B cells, and plasma cells in the lung tissue and sputum. **(A, B)** Representative fluorescence-activated cell sorting (FACS) profiles showing peripheral helper T (Tph) cells, B cells, and plasma cells in the lung tissue (non-rheumatoid arthritis (Non-RA) n=3, rheumatoid arthritis (RA) n=5). Histological samples were evaluated by hematoxylin and eosin (H&E) staining. **(A)** Lung cells isolated from a 48-year-old male patient with Non-RA. This patient had no history of smoking and was diagnosed with lung adenocarcinoma, and there were no background lung abnormalities on chest CT. **(B)** Lung cells isolated from a 79-year-old female patient with RA. This patient was a former smoker and was diagnosed with squamous cell carcinoma of lung and had interstitial lung disease and airway disease. Immunohistochemistry showed T cells (CD3), B cells (CD20), and plasma cells (CD138). Original magnification, x100. **(C–F)** Absolute number of CD4+ T cells **(C)** and Tph cells **(D)** and Tph cell frequencies among CD4+ T cells **(E)** and CD45+ cells **(F)** from lung tissue of patients with non-RA (n=11) and RA (n=6) (**Supplementary Table S3**). Data are expressed as mean ± SEM. **(G)**Tph cells, B cells, and plasma cells in sputum from RA patients (n=7). **(H, I)** Correlation between Tph cells and B cells **(H)** and Tph cells and plasma cells **(I)** in sputum (n=7) and lung (n=8) evaluated by Spearman’s correlation coefficient (**Supplementary Table S4**). **P<0.01 determined by Mann-Whitney test.

Because the number of airway immune cells in a single sputum sample was small, it was difficult to clarify their detailed characteristics or relationships with other immune cells. Therefore, we next examined Tph cells in lung specimens from RA patients who had undergone lung resection due to lung cancer using a separate cohort (**Supplementary Table S3**). As shown in **Figures 4A, B**, and **Supplementary Figure S8**, Tph cells were observed in the lung specimens of RA patients, along with B cells and plasma cells. The frequency of CD4+ T cells in the lungs of RA patients was not different from that of non-RA patients, but the frequency of Tph cells was significantly higher in RA patients (**Figures 4C–F**), and pulmonary Tph cells expressed CCR2 (**Supplementary Figure S9**). Notably, pathological examination revealed infiltration of lymphocytes with follicular-like aggregations in the lungs of RA patients (**Figure 4B**), and IHC showed that the follicular-like aggregates contained T cells and B cells, with a high number of plasma cells in the peripheral areas (**Figure 4B**). B cells and plasma cells were also observed in some sputum samples (**Figure 4G**). In addition, the frequency of Tph cells in sputum and lung correlated with that of B cells (**Figure 4H**), but not with that of plasma cells (**Figure 4I**) (**Supplementary Table S4**).

## 4 Discussion

In this study, we demonstrated Tph cells in the sputum and lungs of RA patients by flow cytometry. These cells expressed CCR2 but not CXCR5, similar to Tph cells in synovial fluid and tissue described in a previous report (1). In addition, we observed local Tph cells together with B cells and plasma cells, with follicular-like aggregations of lymphocytes, in lung specimens from RA patients. Tph cells were originally discovered as a pathological CD4+ T cell subset in the joint tissue of seropositive (positive for RF or ACPA) RA. In the synovium, PD-1^hi^CXCR5^-^CD4^+^ Tph cells, but not PD-1^hi^CXCR5^+^CD4^+^ follicular helper T(Tfh) cells, were the majority in both the lymphoid aggregation site and the diffuse lymphoid infiltration site. Tph cells are located adjacent to B cells and can help differentiate B cells into plasma cells. These cells produce IL-21 and CXCL13, which stimulate B cell survival, differentiation, and aggregation in the local site. Although we could not investigate the direct role of lung Tph cells on B cells, there was a positive correlation between sputum IgG levels and number of sputum Tph cells (r=0.504, p=0.012), suggesting the involvement of lung Tph cells in local antibody production.

Accumulating evidence for individuals at high risk of future RA development has indicated the importance of mucosal inflammatory processes in the development of systemic immune alterations and arthritis, termed the ‘mucosal origins hypothesis’ (25–27). The lung, along with the periodontal, intestinal, and cervicovaginal mucosa, has attracted attention as a site of ACPA production. Willis et al. first reported that autoantibodies were detectable in the sputum of 39% of at-risk RA but seronegative subjects, 65% of at-risk RA and seropositive subjects, and 86% of subjects with early RA (20). In the at-risk group, the ratio of autoantibodies to total Ig was higher in sputum than in serum, suggesting that in some cases RA-associated autoantibodies were generated in the lungs before the appearance of joint symptoms. Analysis of bronchial biopsies and bronchoalveolar lavage (BAL) in early RA without apparent lung diseases showed that lymphocyte infiltration was more frequently observed in ACPA-positive patients compared with ACPA-negative patients (28). In addition, B cells and plasma cells were found only in ACPA-positive patients together with germinal center-like structures in bronchial biopsies. Although most of our study subjects had relatively long disease and treatment histories and had ILD and/or AD, the mucosal inflammation may persist throughout the life of RA patients. Furthermore, sputum Tph cells, but not blood Tph cells, were increased in the patients with RF-positive and high IgG levels, suggesting that the airways are involved in systemic inflammation in RA patients.

In our study, sputum Tph cells tended to be increased in RA patients with ILD and/or AD and were significantly increased in patients with AD. Of note, the frequency of blood Tph cells was not affected by the presence or absence of ILD and/or AD, and there was no correlation between sputum Tph cells and blood Tph cells, suggesting that airway Tph cells may be a unique biomarker or therapeutic target. Indeed, ectopic lymphoid-like structures named inducible bronchus-associated lymphoid tissue (iBALT) are well-known in RA lung complications (29). Sato et al. reported that iBALT was observed in follicular bronchitis in RA patients (30), and Rangel-Moreno et al. reported that iBALT was specifically found in RA-associated interstitial pneumonia and Sjogren’s syndrome-associated interstitial pneumonia (31). In the analysis of two large prospective cohorts in the US, elevated ACPA prior to RA onset was shown to be a risk factor for COPD and asthma (32, 33). Although it is not clear whether iBALT or ACPA production is a cause or a consequence of lung/airway disease, these observations support the concept of a strong relationship between the lung/airway as a site of antibody production and RA as a systemic disease. As shown in **Figure 3**, sputum Tph cells were increased in AD without an increase in airway neutrophils and lymphocytes. Furthermore, the frequency of sputum Tph cells did not differ according to sputum bacterial culture results, chest CT findings, FeNO, pulmonary symptoms, or lung function. Therefore, Tph cells appear to be associated with the presence of AD, but not with the severity of AD. In contrast, sputum Tph cells were elevated in patients with high DAS28-ESR and tended to be elevated in patients with high DAS28-CRP. These data suggest that airway Tph cells may be a new research target for understanding the relationship between RA lung/airway lesions and joint inflammation.

This study has several limitations. First, this is a single-center, small-group study. The patients analyzed in this study were recruited from the respiratory outpatient clinic. Consequently, the percentages of patients with lung/airway involvement were much higher than those in the general RA population. Indeed, the number of non-ILD and/or AD patients eligible for sputum analysis was very small. However, this may be a technical limitation due to the difficulty in collecting sufficient samples for evaluation from patients without pulmonary symptoms (**Supplementary Figure S1**). Furthermore, the number of patients in the anti-CCP2 negative or RF negative groups was also small. This may be due to the relationship between autoantibodies and lung/airway diseases in RA patients. Several reports have shown that RA-related lung abnormalities (bronchiectasis, bronchiolitis, interstitial lung disease, etc.) are more common in seropositive RA (34, 35). Second, most of the patients were treated with DMARDs and/or glucocorticoids, which may have affected the results. However, we observed increased sputum Tph cells in the patients with high RA disease activity even when receiving conventional treatment from rheumatologists. This may indicate that the regulation of airway Tph cells, which are involved in B cell activation and local antibody production, may be a novel therapeutic strategy. Third, we did not investigate the direct role of sputum or lung Tph cells on B cells and other immune cells, or the detailed phenotype and possible function of Tph cells involved in RA disease modification. In addition, the relationship between iBALT and Tph cells was not demonstrated in this study. Further research is needed to elucidate the pathological roles of local Tph cells in RA patients with lung/airway involvement.

## 5 Conclusion

In conclusion, we identified Tph cells in the sputum as well as in the synovium of RA patients. Sputum Tph cells were associated with disease severity in RA patients on treatment, but not with severity of airway disease. They may represent a novel target for disease management and treatment.

## Data Availability

The raw data supporting the conclusions of this article will be made available by the authors, without undue reservation.

## Supplementary material

Supplementary Figure 1 Patient flow of the 51 patients enrolled in this study, sputum samples were collected and analyzed in 40 patients. Blood samples were collected at the same time in 38 patients. AD, airway disease; ILD, interstitial lung disease; Non-ILD/AD, rheumatoid arthritis without apparent lung/airway disease.

Supplementary Figure 2 Representative chest computed tomography (CT) findings. **(A)** Honeycomb, **(B)** Reticular opacities, **(C)** Traction bronchiectasis, **(D)** Ground-glass opacities, **(E)** Bronchiectasis, **(F)** Granular shadow, **(G)** Mucus plug.

Supplementary Figure 3 Fluorescence minus one (FMO) control to determine fluorescence spread into the PE channel. Fluorescence spread into the PE channel was assessed by FMO using blood from healthy controls (n=3).

Supplementary Figure 4 Gating strategy for peripheral helper T cells in synovial fluid, synovial tissue, and sputum. Representative data of 5 synovial fluid samples, 5 synovial tissue samples, and 40 sputum samples.

Supplementary Figure 5 Frequency of sputum peripheral helper T cells according to anti-CCP2 antibody results. Sputum peripheral helper T (Tph) cells from rheumatoid arthritis patients divided by the positive (n=35) or negative (n=3) for anti-CCP2 antibody. Data are expressed as mean ± SEM. P values were determined by Mann-Whitney test.

Supplementary Figure 6 Correlation between sputum peripheral helper T cells and IgG levels. Spearman’s correlation coefficient for sputum peripheral helper T (Tph) cells and blood IgG levels **(A)** and sputum Tph cells and sputum IgG levels **(B)**.

Supplementary Figure 7 Peripheral helper T cells in airway disease without interstitial lung disease. **(A)** Sputum from rheumatoid arthritis (RA) patients with airway disease (AD) without interstitial lung disease (ILD) was evaluated. Non-ILD/AD indicates RA without apparent lung/airway disease. **(B)** Total cell counts, percentages of neutrophils and lymphocytes in the sputum of RA patients with AD without ILD (Non-ILD/AD n=4, AD n=13-17). **(C)** Total number and percentage of peripheral helper T (Tph) cells from the sputum of RA patients with AD without ILD (Non-ILD/AD n=4, AD n=16-17). **(D)** Sputum Tph cells based on the positive or negative general sputum culture (sputum bacteria) (positive n=9, negative n=8) and acid-fast bacilli culture (nontuberculous mycobacteria: NTM) (positive n=7, negative n=10). **(E–I)** Sputum Tph cells divided by chest computed tomography (CT) findings (plugging (≤4 n=6, >4 n=11), bronchiectasis (≤2 n=7, >2 n=8), and granular shadow (≤2 n=8, >2 n=8)) **(E)**, the value of exhaled nitric oxide fraction (FeNO) (<15 ppb n=8, ≥15ppb n=6) **(F)**, COPD assessment test (CAT) score (≤10 n=10, >10 n=7) and FEV_1_/FVC% (≥ 70% n=10, <70% n=5) **(G)**, blood IgG levels (<1300 mg/dL n=9, ≥ 1300 mg/dL n=7) and sputum IgG levels (<2.3 mg/dL n=6, ≥ 2.3 mg/dL n=10) **(H)**, CRP (≤0.3 mg/dL n=9, >0.3 mg/dL n=8) and ESR (≤22 mm/h n=8, >22 mm/h n=9) **(I)**, and DAS-CRP (≤Low n=9, ≥Moderate n=7) and DAS28-ESR (≤Low n=8, ≥Moderate n=8). Disease activity based on DAS28-CRP (remission, <2.3; low disease activity, ≥2.3 and <2.7; moderate disease activity, ≥2.7 and ≤4.1; and high disease activity, >4.1) and on DAS28-ESR (remission, <2.6; low disease activity, ≥2.6 and <3.2; moderate disease activity, ≥3.2 and ≤5.1; and high disease activity, >5.1) **(J)**. Blood and sputum IgG and FeNO cutoff values were determined by the median of all subjects. Data are shown as mean ± SEM. *P<0.05, **P<0.01 determined by Mann-Whitney test.

Supplementary Figure 8 Gating strategy for B cells and plasma cells in sputum and peripheral helper T cells, B cells, and plasma cells in the lung. Representative data of 7 sputum samples and 8 lung samples.

Supplementary Figure 9 CCR2 expression on peripheral helper T cells. Representative data of 9 sputum samples and 3 lung samples. Control, PD1^-^CD4^+^ T cells.
