# Supplementary figures and images for: Increased sputum peripheral helper T cells are associated with the severity of rheumatoid arthritis but not with the severity of airway disease
Source: Front Immunol. 2025 Feb 27;16:1526881. doi: 10.3389/fimmu.2025.1526881 (PMC11903478; doi:10.3389/fimmu.2025.1526881)

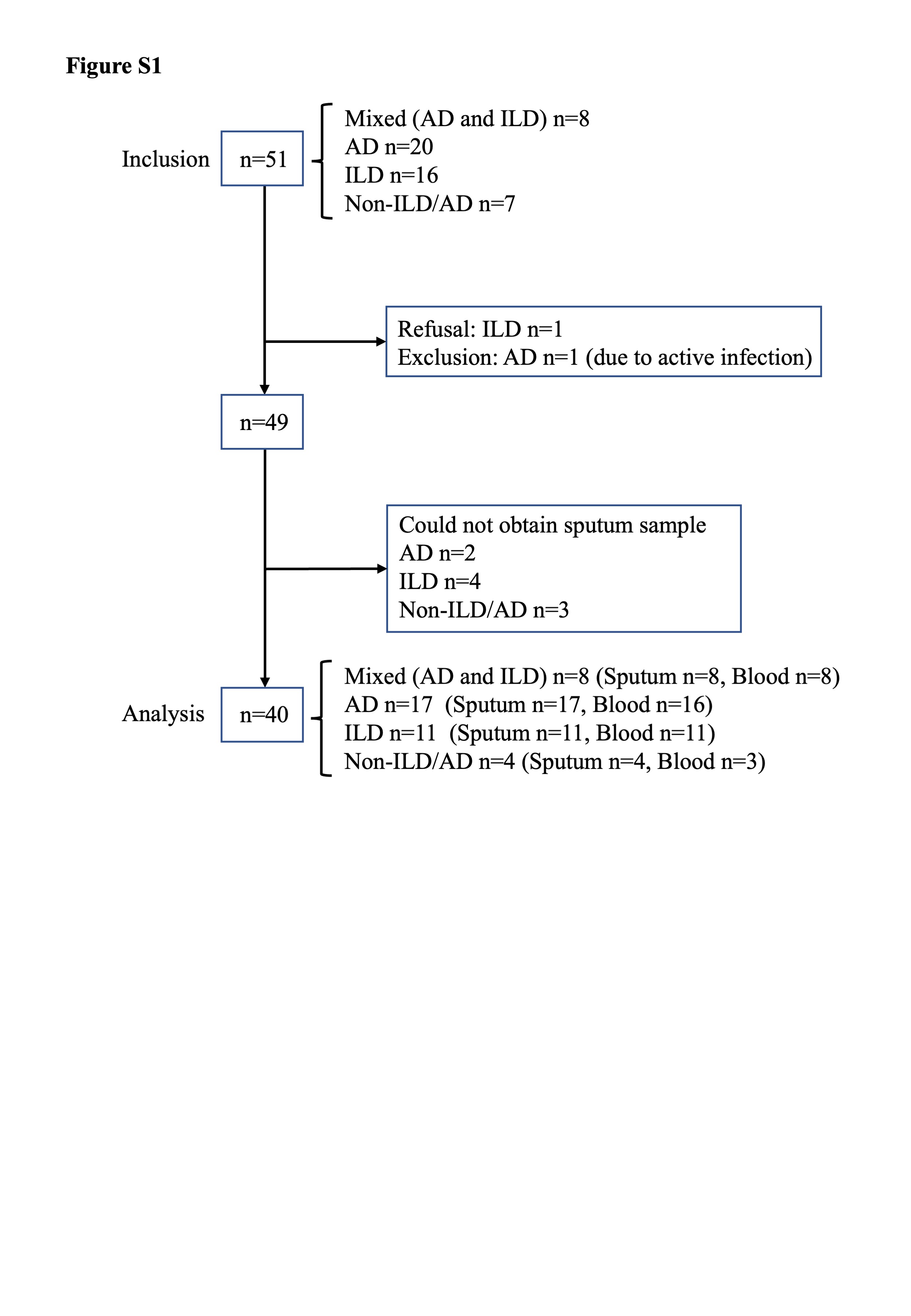

Supplement: Supplementary Figure 1 — Patient flow of the 51 patients enrolled in this study, sputum samples were collected and analyzed in 40 patients. Blood samples were collected at the same time in 38 patients. AD, airway disease; ILD, interstitial lung disease; Non-ILD/AD, rheumatoid arthritis without apparent lung/airway disease. [file Image1.jpeg]

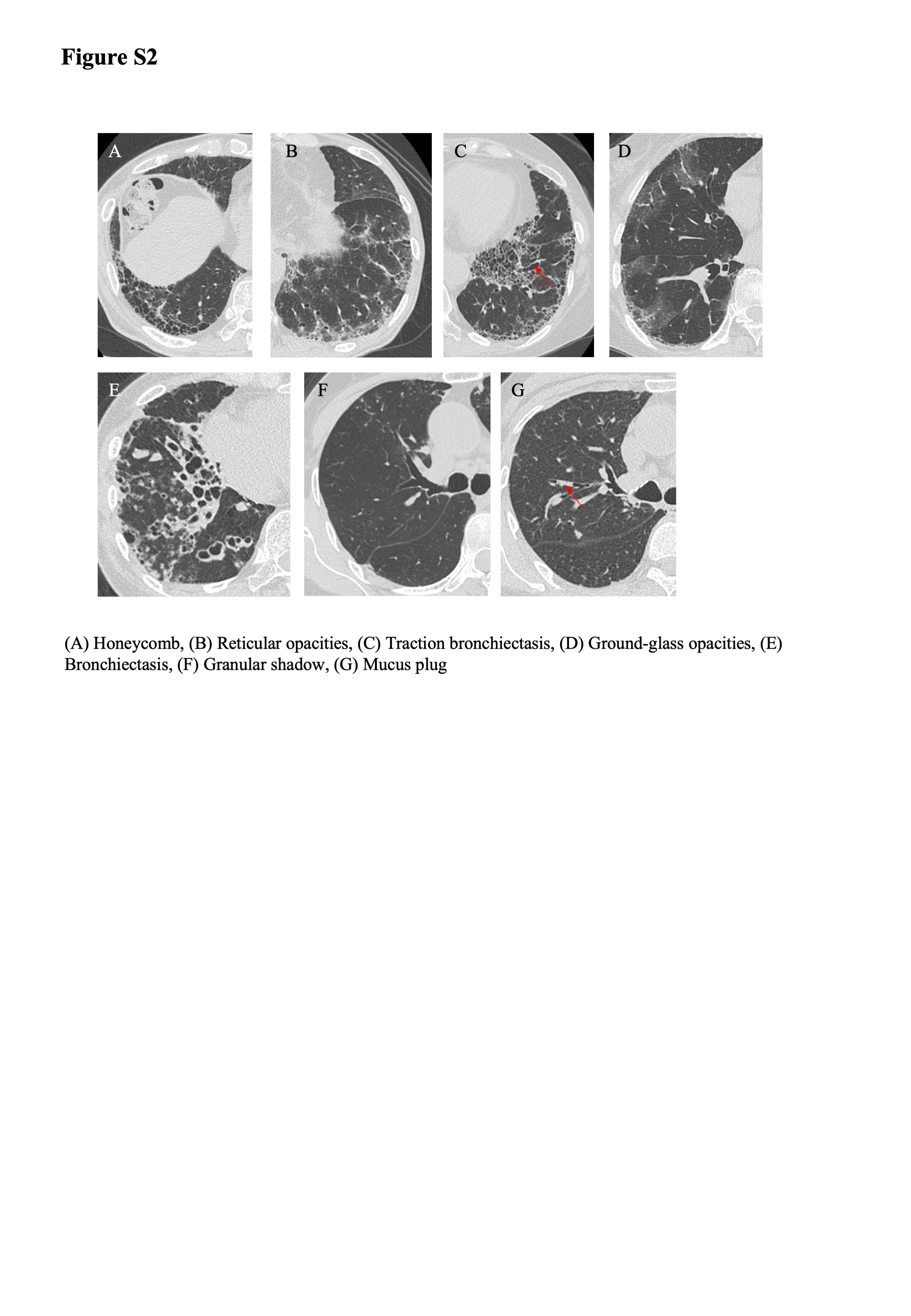

Supplement: Supplementary Figure 2 — Representative chest computed tomography (CT) findings. (A) Honeycomb, (B) Reticular opacities, (C) Traction bronchiectasis, (D) Ground-glass opacities, (E) Bronchiectasis, (F) Granular shadow, (G) Mucus plug. [file Image2.jpeg]

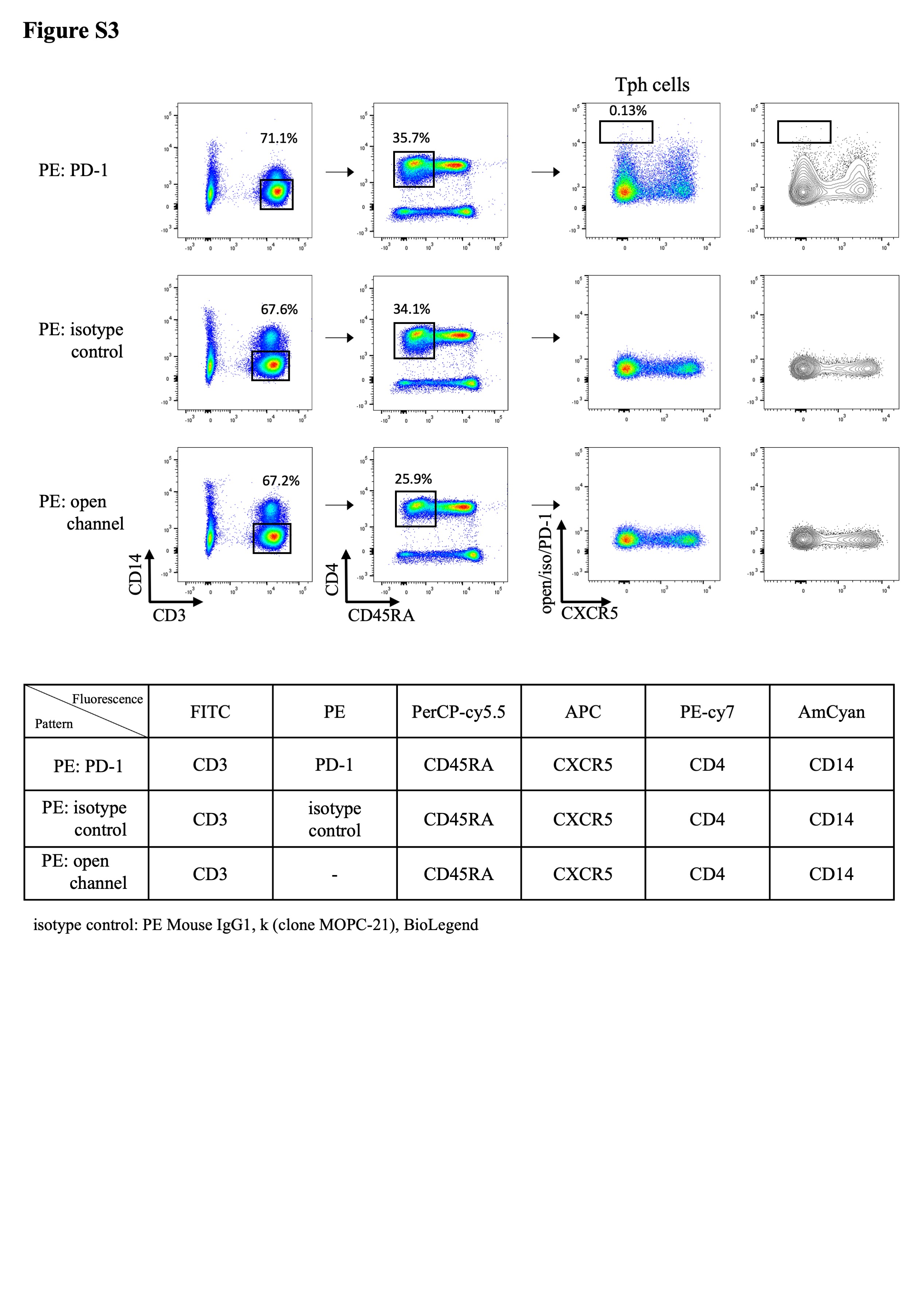

Supplement: Supplementary Figure 3 — Fluorescence minus one (FMO) control to determine fluorescence spread into the PE channel. Fluorescence spread into the PE channel was assessed by FMO using blood from healthy controls (n=3). [file Image3.jpeg]

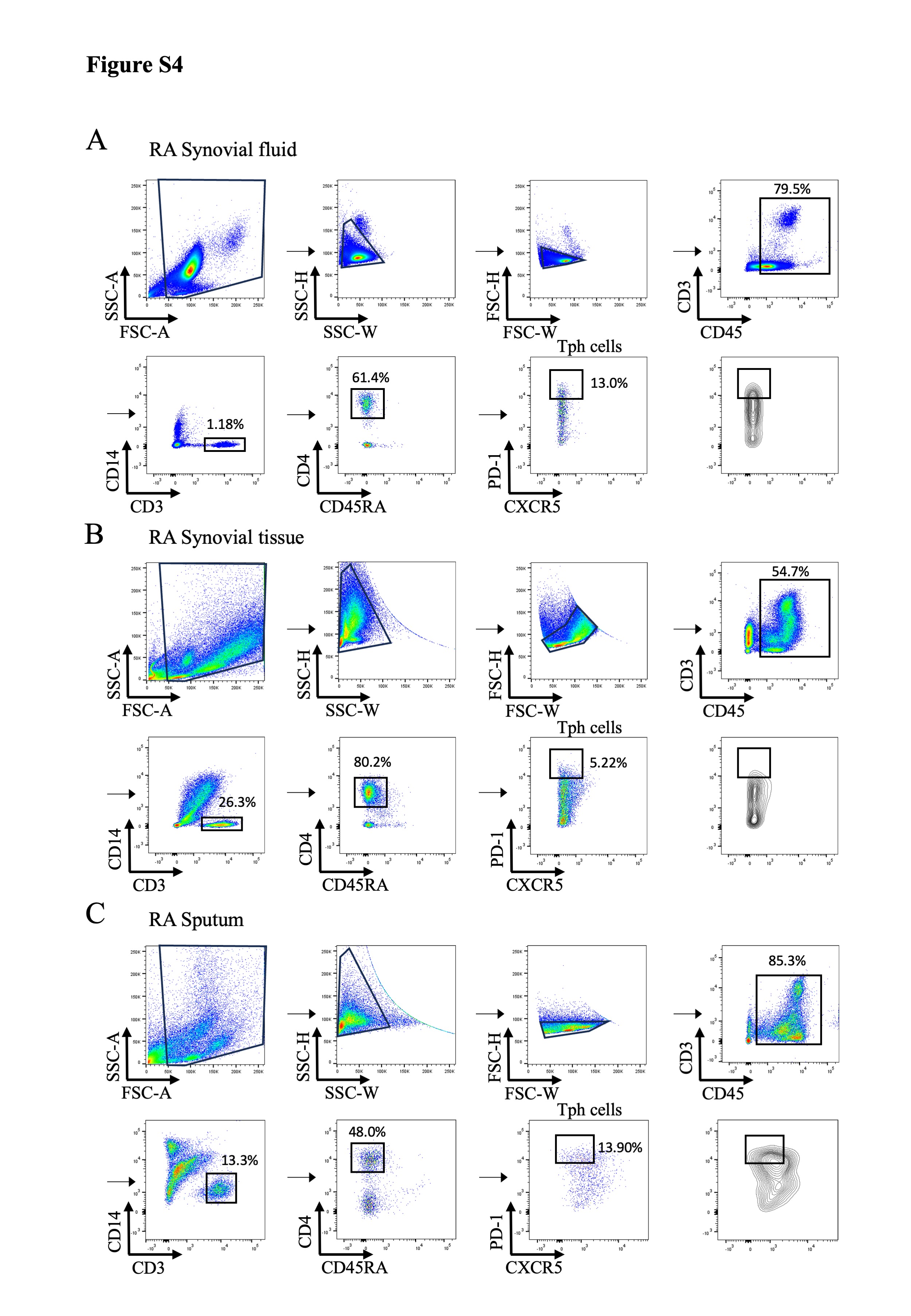

Supplement: Supplementary Figure 4 — Gating strategy for peripheral helper T cells in synovial fluid, synovial tissue, and sputum. Representative data of 5 synovial fluid samples, 5 synovial tissue samples, and 40 sputum samples. [file Image4.jpeg]

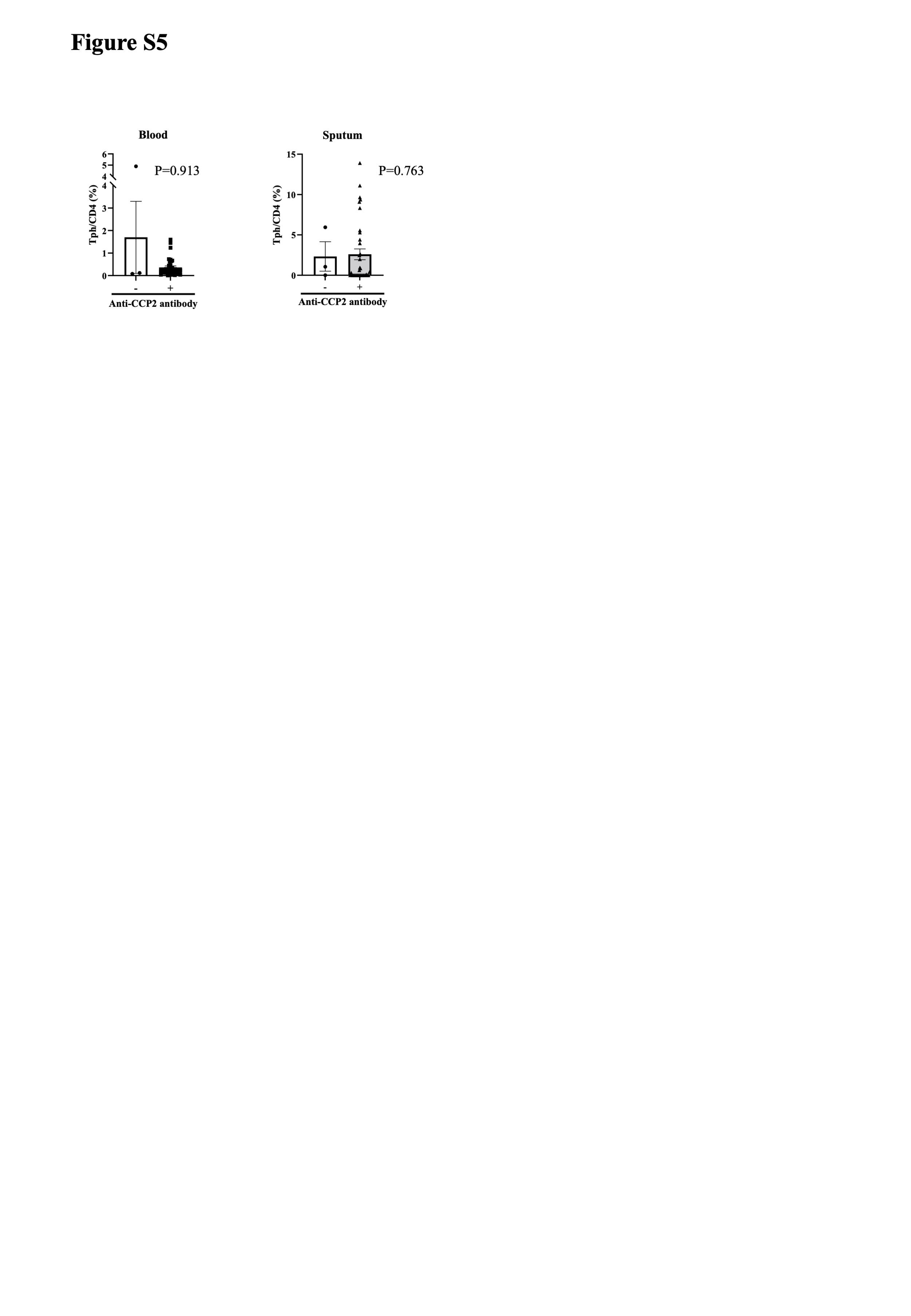

Supplement: Supplementary Figure 5 — Frequency of sputum peripheral helper T cells according to anti-CCP2 antibody results. Sputum peripheral helper T (Tph) cells from rheumatoid arthritis patients divided by the positive (n=35) or negative (n=3) for anti-CCP2 antibody. Data are expressed as mean ± SEM. P values were determined by Mann-Whitney test. [file Image5.jpeg]

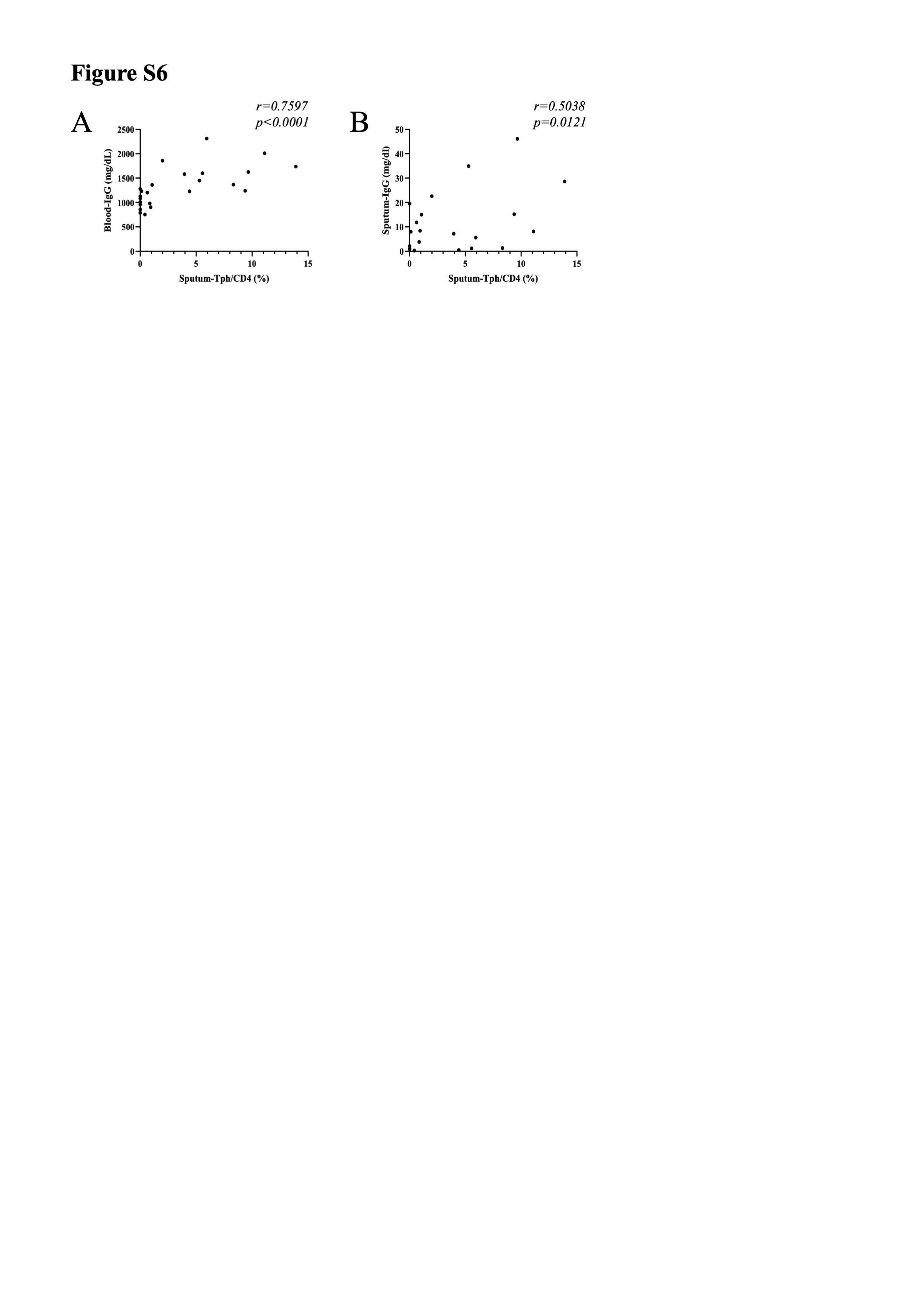

Supplement: Supplementary Figure 6 — Correlation between sputum peripheral helper T cells and IgG levels. Spearman’s correlation coefficient for sputum peripheral helper T (Tph) cells and blood IgG levels (A) and sputum Tph cells and sputum IgG levels (B). [file Image6.jpeg]

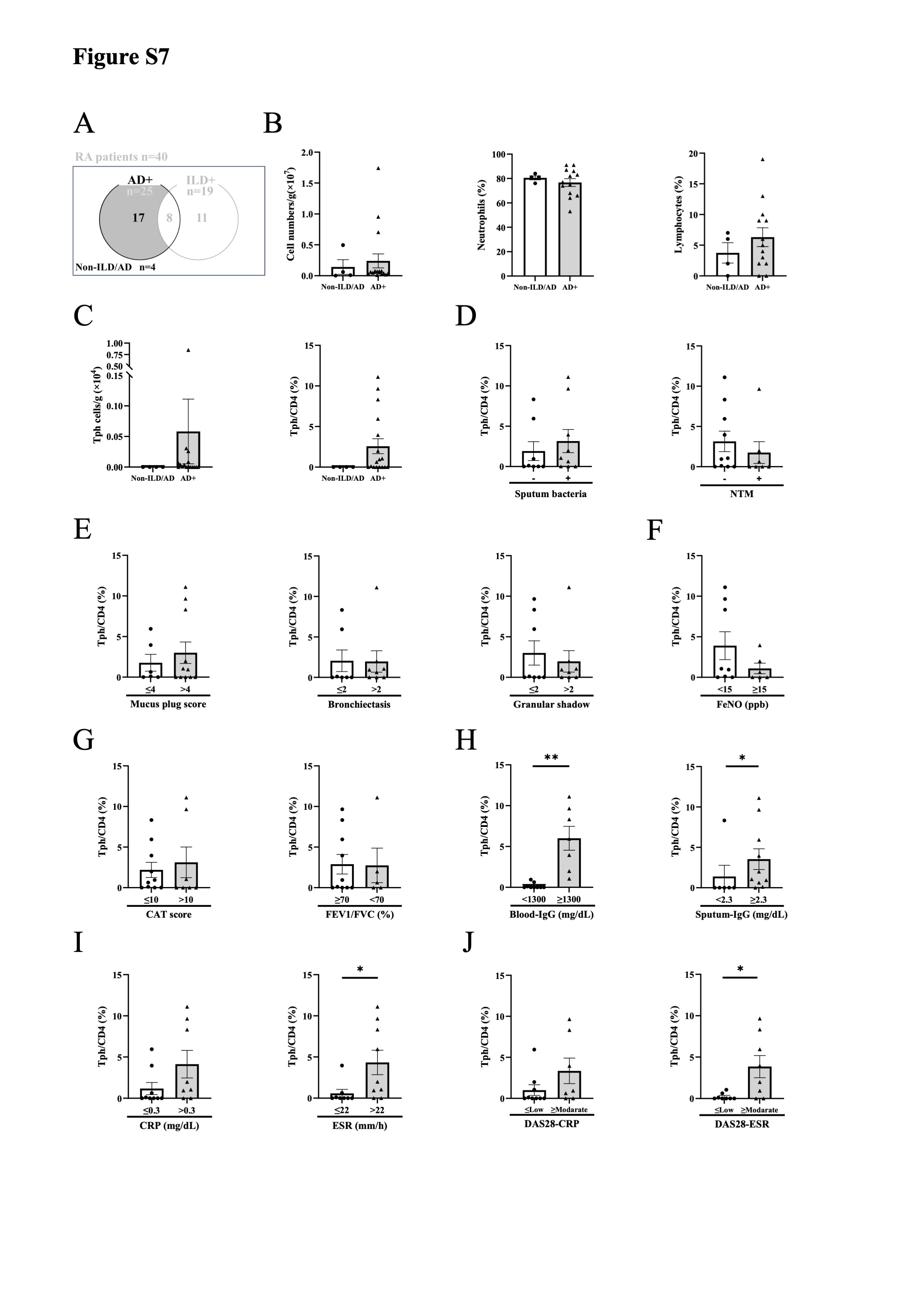

Supplement: Supplementary Figure 7 — Peripheral helper T cells in airway disease without interstitial lung disease. (A) Sputum from rheumatoid arthritis (RA) patients with airway disease (AD) without interstitial lung disease (ILD) was evaluated. Non-ILD/AD indicates RA without apparent lung/airway disease. (B) Total cell counts, percentages of neutrophils and lymphocytes in the sputum of RA patients with AD without ILD (Non-ILD/AD n=4, AD n=13-17). (C) Total number and percentage of peripheral helper T (Tph) cells from the sputum of RA patients with AD without ILD (Non-ILD/AD n=4, AD n=16-17). (D) Sputum Tph cells based on the positive or negative general sputum culture (sputum bacteria) (positive n=9, negative n=8) and acid-fast bacilli culture (nontuberculous mycobacteria: NTM) (positive n=7, negative n=10). (E–I) Sputum Tph cells divided by chest computed tomography (CT) findings (plugging (≤4 n=6, >4 n=11), bronchiectasis (≤2 n=7, >2 n=8), and granular shadow (≤2 n=8, >2 n=8)) (E), the value of exhaled nitric oxide fraction (FeNO) (<15 ppb n=8, ≥15ppb n=6) (F), COPD assessment test (CAT) score (≤10 n=10, >10 n=7) and FEV1/FVC% (≥ 70% n=10, <70% n=5) (G), blood IgG levels (<1300 mg/dL n=9, ≥ 1300 mg/dL n=7) and sputum IgG levels (<2.3 mg/dL n=6, ≥ 2.3 mg/dL n=10) (H), CRP (≤0.3 mg/dL n=9, >0.3 mg/dL n=8) and ESR (≤22 mm/h n=8, >22 mm/h n=9) (I), and DAS-CRP (≤Low n=9, ≥Moderate n=7) and DAS28-ESR (≤Low n=8, ≥Moderate n=8). Disease activity based on DAS28-CRP (remission, <2.3; low disease activity, ≥2.3 and <2.7; moderate disease activity, ≥2.7 and ≤4.1; and high disease activity, >4.1) and on DAS28-ESR (remission, <2.6; low disease activity, ≥2.6 and <3.2; moderate disease activity, ≥3.2 and ≤5.1; and high disease activity, >5.1) (J). Blood and sputum IgG and FeNO cutoff values were determined by the median of all subjects. Data are shown as mean ± SEM. *P<0.05, **P<0.01 determined by Mann-Whitney test. [file Image7.jpeg]

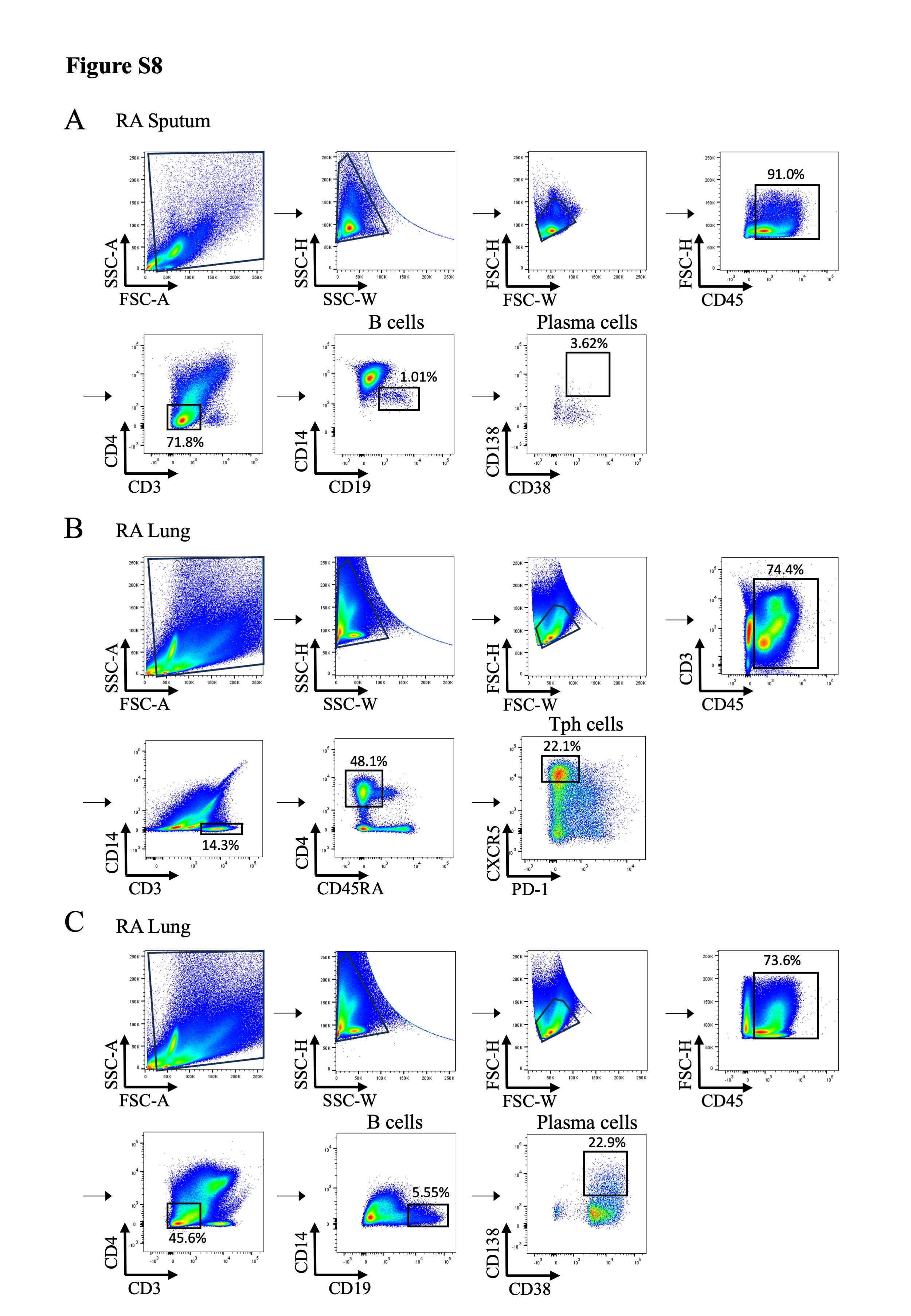

Supplement: Supplementary Figure 8 — Gating strategy for B cells and plasma cells in sputum and peripheral helper T cells, B cells, and plasma cells in the lung. Representative data of 7 sputum samples and 8 lung samples. [file Image8.jpeg]

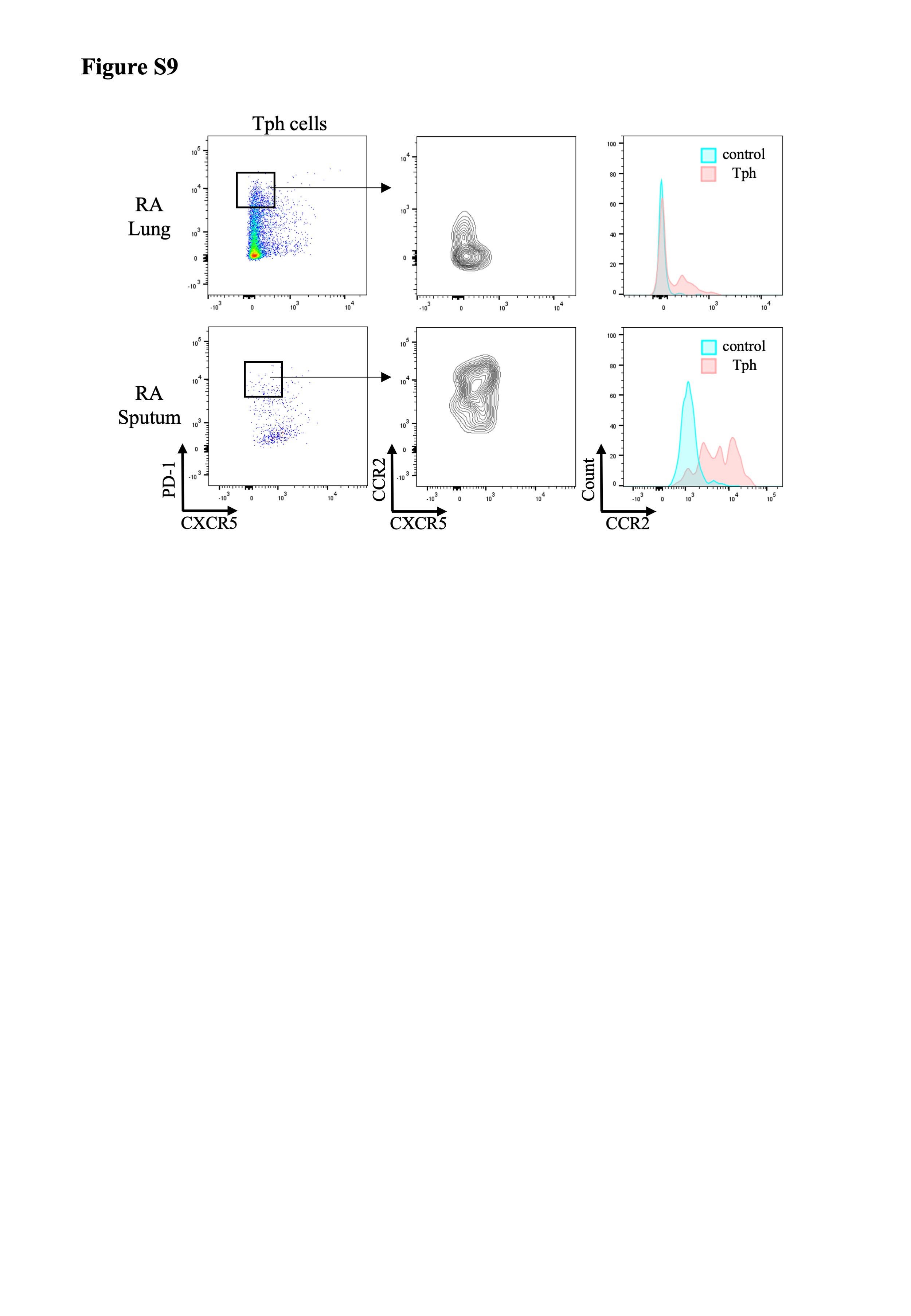

Supplement: Supplementary Figure 9 — CCR2 expression on peripheral helper T cells. Representative data of 9 sputum samples and 3 lung samples. Control, PD1-CD4+ T cells. [file Image9.jpeg]
