# Supplementary material for: Increased sputum peripheral helper T cells are associated with the severity of rheumatoid arthritis but not with the severity of airway disease
Source: Front Immunol. 2025 Feb 27;16:1526881. doi: 10.3389/fimmu.2025.1526881 (PMC11903478; doi:10.3389/fimmu.2025.1526881)
Supplement: Supplementary file 10 [file DataSheet1.pdf]

Table S1. Definition of chest computed tomography (CT) findings

| CT findings | Definition | Score |
|---|---|---|
| Interstitial pneumonia | ・Subpleural honeycombing<br>・Reticular opacities<br>・Traction bronchiectasis<br>・Bilateral patchy ground-glass opacities | Positive if any of these findings are present |
| Bronchiectasis | Bronchial diameter clearly larger than the accompanying pulmonary artery | Evaluated according to the number of pulmonary lobes and segments affected, with the lingula and middle lobe considered as independent lobes (0-6) [1] |
| Granular shadow | Cluster of small shadows of 5 mm or less | Same as above |
| Mucus plugs | Areas of opacification within the airway lumen, contiguous with patent airway lumen across sequential transverse CT slices | Number of bronchopulmonary segments with ≧1 mucus plug (s) and summed (0-18) [2] |

CT, computed tomography.

[1] Martínez-García MÁ, et al. Eur Respir J. 2014 May; 43(5): 1357-67.

[2] Okajima Y, et al. Chest.2020; 158(1): 121-130.

Table S2. Patient characteristics of synovial tissue and synovial fluid analysis

Study population n=5

| | RA |
|---|---|
| Patients, n | 5 |
| Age, median (range) years | 69 (62-73) |
| Female, n (%) | 3 (60.0) |
| Duration of RA (years) | 15.6±5.41 |
| Smoking history(never/ex/current), n | 2/2/1 |
| Pack-years | 14.0±18.12 |
| DAS28-CRP | 2.68±0.361 |
| Disease activity (remission/low/moderate/high), n | 1/2/2/0 |
| RF (positive/negative), n | 3/2 |
| Treatment chraracteristics | |
| Use of oral glucocorticoids, n (%) | 2 (40.0) |
| Use of methotorexate, n (%) | 3 (60.0) |
| Use of bDMARDs, n (%) | 2 (40.0) |

RA, Rheumatoid arthritis; DAS28, 28-joint disease activity score; RF, rheumatoid factor; bDMARDs, biological disease-modifying antirheumatic drugs.

Disease activity was classified based on DAS28-CRP (remission, <2.3; low disease activity, ≥2.3 and <2.7; moderate disease activity, ≥2.7 and ≤4.1; and high disease activity, >4.1).

Data presented as n (%) or mean±SD unless otherwise noted.

Table S3. Patient characteristics of lung tissue analysis

Study population n=17

| | Non-RA | RA |
|---|---|---|
| Patients, n | 11 | 6 |
| Age, median (range) years | 65 (48-83) | 75.5 (65-79) |
| Female, n (%) | 3 (27.3) | 3 (50.0) |
| Smoking history(never/ex/current), n | 4/7/0 | 0/6/0 |
| Pack-years | 32.45±28.51 | 38.5±17.72 |
| Treatment chraracteristics | | |
| Oral steroid, n (%) | 0 | 3 (50) |
| Methotorexate, n (%) | 0 | 4 (66.7) |
| Biologic agent, n (%) | 0 | 1 (16.7) |
| Chest CT | | |
| Pattern (normal/ILD/airway lesion/mixed), n | 4/0/7/0 | 4/0/1/1 |
| Lung function test | | |
| VC % predicted (%) | 113.44±13.36 | 92.48±10.01* |
| $FEV_1$ % predicted (%) | 97.10±13.20 | 94.33±10.15 |
| $FEV_1$/FVC % (%) | 69.89±10.36 | 78.85±8.68 |
| Complications | | |
| Asthma, n (%) | 0 | 0 |
| COPD, n (%) | 4 (36.3) | 1 (16.7) |
| Pathology of lung cancer | | |
| AD, n (%) | 8 (72.7) | 3 (50.0) |
| SQ, n (%) | 2 (18.1) | 3 (50.0) |
| LCNEC, n (%) | 1 (9.1) | 0 |

RA, Rheumatoid arthritis; VC, vital capacity; $FEV_1$, forced expiratory volume in one second; COPD, chorionic obstructive pulmonary disease; AD, adenocarcinoma; SQ, squamous carcinoma; LCNEC, large cell neuroendocrine carcinoma.

Data presented as n (%) or mean±SD unless otherwise noted.

*P<0.05 vs. the Non-RA, determined by Mann Whitney U test.

Table S4. Patient characteristics for B cells and plasma cells analysis

Study population n=15

| | Sputum | Lung |
|---|---|---|
| Patients, n | 7 | 8 |
| Age, median (range) years | 52 (37-58) | 73.5 (48-79) |
| Female, n (%) | 4 (57.1) | 4 (50.0) |
| RA | 7 | 5 (62.5) |
| Smoking history(never/ex/current), n | 4/3/0 | 2/6/0 |
| Pack-years | 27.00±37.77 | 26.75±21.58 |
| Treatment chraracteristics | | |
| Oral steroid, n (%) | 3 (42.9) | 3 (37.5) |
| Methotorexate, n (%) | 5 (71.4) | 3 (37.5) |
| Biologic agent, n (%) | 2 (28.6) | 1 (0.125) |
| Chest CT | | |
| Pattern (normal/ILD/airway lesion/mixed), n | 0/2/4/1 | 6/0/1/1 |
| Lung function test | | |
| VC % predicted (%) | 101.36±17.58 | 100.38±14.81 |
| $FEV_1$ % predicted (%) | 97.80±25.97 | 96.33±10.10 |
| $FEV_1$/FVC % (%) | 72.56±10.46 | 76.81±8.88 |
| Complications | | |
| Asthma, n (%) | 1 (14.3) | 0 |
| COPD, n (%) | 0 | 2 (25.0) |
| Pathology of lung cancer | | |
| AD, n (%) | - | 3 (37.5) |
| SQ, n (%) | - | 5 (62.5) |
| LCNEC, n (%) | - | 0 |

RA, Rheumatoid arthritis; VC, vital capacity; $FEV_1$, forced expiratory volume in one second; COPD, chorionic obstructive pulmonary disease; AD, adenocarcinoma; SQ, squamous carcinoma; LCNEC, large cell neuroendocrine carcinoma.
